# Realising the potential of radioligand therapy: policy solutions for the barriers to implementation across Europe

**DOI:** 10.1007/s00259-020-04745-7

**Published:** 2020-03-13

**Authors:** C. Merkel, C. H. Whicher, J. Bomanji, K. Herrmann, J. Ćwikła, N. Jervis, S. Wait, A. Chiti

**Affiliations:** 1 The Health Policy Partnership, London, UK; 2 Institute of Nuclear Medicine UCLH NHS Foundation Trust, London, UK; 3 Clinic for Nuclear Medicine, Essen University Hospital, Essen, Germany; 4 Department of Cardiology and Cardiac Surgery, Medical School University of Warmia and Mazury, Olsztyn, Poland; 5 Diagnostic and Therapy Center, Warsaw, Poland; 6 Neuroendocrine Cancer UK, Leamington Spa, UK; 7 Department of Biomedical Sciences, Humanitas University, Milan, Italy; 8 Unit of Nuclear Medicine, Humanitas Research Hospital, Milan, Italy

## Background

The use of targeted radiation with radioisotopes has been applied for decades in thyroid cancer. However, the use of radioligand therapy—also referred to as peptide-receptor radionuclide therapy (PRRT), systemic radiation therapy, targeted radionuclide therapy (TRT), targeted radiotherapy or molecular radiotherapy—is now gaining ground more broadly in oncology. Therapies have been approved for a small number of cancers where few treatment options exist, such as midgut neuroendocrine tumours and metastatic castration-resistant prostate cancer (mCRPC) [1, 2], and they have been shown to improve progression-free survival and quality of life for many patients [3–7]. The approach is also being explored in other cancer and non-cancer conditions.

Nuclear medicine has, traditionally, sat somewhat on the sidelines of cancer care; however, the evolving oncology landscape suggests its role will grow, with broader applications in both diagnostics and therapy [8]. Nuclear medicine specialists will need to be fully integrated into multidisciplinary cancer care teams, and appropriate resourcing to deliver radioligand therapy—in terms of hospital infrastructure and workforce, as well as nuclear waste facilities—will need to be taken into account in future cancer plans and care pathways.

Over the course of 2019, we conducted desk research and semi-structured expert interviews to explore potential barriers to achieving this integration in practice, drawing on insights from five different countries—Germany, Italy, Poland, Spain and the United Kingdom. The resulting document, ‘Radioligand therapy: realising the potential of targeted cancer care’, [9] was launched at the European Parliament in January 2020 along with an accompanying video explaining radioligand therapy for a lay audience. This report is an important reminder to the fields of nuclear medicine and oncology of the factors necessary to ensure advances in nuclear medicine reach patients in future models of cancer care.

## What are the potential barriers to integration of radioligand therapy into cancer care?

Healthcare systems are often inadequately prepared for greater utilisation and integration of radioligand therapy. The report identified six key barriers to the greater integration of radioligand therapy into cancer care: low awareness and understanding; limited professional capacity, training and workforce planning; unclear models of care; inadequate physical capacity and resourcing in hospitals; evolving legislation, regulation and policy; and lack of data and research [9].

### Low awareness and understanding

Many oncologists and other clinicians engaged in cancer care may not fully understand what radioligand therapy is. Moreover, patients—and sometimes clinicians too—may be wary due to negative preconceptions around the use of radioactive substances [10, 11].

### Limited professional capacity, training and workforce planning

Different countries, and even different hospitals in the same country, often have disparate ways of organising the delivery of radioligand therapy [12]. Generally, there are few healthcare personnel appropriately trained in this treatment approach [10], restricting it to a small number of specialist centres.

Despite being a tenet of most clinical guidelines and cancer plans, multidisciplinary working is not always implemented in practice. Roles and responsibilities of different members of the multidisciplinary team can be unclear [11, 13]; this holds true particularly for the inclusion of nuclear medicine specialists in tumour boards in non-specialist hospitals. In some centres, the limited number of nuclear medicine specialists may simply mean there is not enough capacity for them to participate in every multidisciplinary tumour board.

Educating all members of the multidisciplinary cancer team on radioligand therapy is important, as mentioned above; however, there seem to be few consistent educational initiatives appropriate to the whole cancer care team.

### Unclear models of care

The provision of radioligand therapy requires intensive planning with clear workflows and processes [10, 14, 15]. New processes around radioligand therapy may be disruptive to current cancer care pathways, which may be a barrier in itself [10]. Greater harmonisation of guidelines and treatment protocols is also a challenge with radioligand therapy, causing inconsistencies in practice. As the evidence base evolves, both guidelines and models of care need to be updated in line with the latest scientific advances [16, 17].

### Inadequate physical capacity and resourcing in hospitals

There are significant geographical variations in access to radioligand therapy, meaning people often travel significant distances, and even across countries, for treatment [10]. The approach is frequently provided as an inpatient procedure, which may require isolation of patients in lead-lined rooms in line with local legislation—and existing treatment centres may not have sufficient capacity to meet patient needs. There may be also additional requirements for equipment or storage facilities for contaminated materials. In some centres, moving to an outpatient model is being explored to potentially alleviate some of these capacity issues, as not all radiopharmaceuticals require a strict hospital regime.

As demand for all types of radioisotopes continues to grow, sustained efforts are needed from both the health and energy sectors to ensure consistent supply and delivery. Most medical radioisotopes are created in a small number of nuclear reactors, which are becoming increasingly unreliable due to old age [18–20]. There can be additional logistical difficulties in post-production processing and distribution to hospitals [18–21]. Such unpredictability in the global supply chain has directly impacted availability of diagnostic tests and medical procedures involving certain radioisotopes [18, 20].

### Evolving legislation, regulation and policy

Regulatory frameworks for approval of radioligand therapy are inconsistent between countries—in some instances radioligands are considered pharmaceuticals, in others they are classified and regulated as radioactive substances. Furthermore, regulatory frameworks do not fully account for differences between radioisotopes, for example, between diagnostic radioisotopes and therapeutic radioisotopes in which alpha or beta radiation is the main energy carrier. Such rigid frameworks can restrict the use of certain types of radioligand therapy and affect who provides treatment and how [11, 15, 22]. Both international and national regulatory frameworks developed for conventional medicines may need to be adapted to be appropriate for the evaluation of radioligand therapy and radioisotopes [23].

Waste disposal policies also need careful planning, as different radioisotopes require different processes [14]. Although some radioisotopes do not require significant specialised waste collection or storage, these processes may be required in other cases; a ‘one size fits all’ nuclear waste disposal policy is therefore not appropriate. As demand increases, there may be additional pressures on such processes.

### Lack of data and research

The limited availability of representative clinical data on radioligand therapy poses a challenge [12], potentially contributing to significant disparities in availability across Europe [10, 24]. The absence of clear and consistent understanding of what constitutes a response to radioligand therapy is an important challenge [5, 12, 24, 25]. Analysis of existing clinical trial data may be hindered by the heterogeneity of patient groups with advanced cancer and the retrospective nature of the data [5, 12]. In the field of neuroendocrine tumours, the low number of people affected presents an additional barrier. More real-world data reflecting the longer-term experience of patients on radioligand therapy is needed to improve our understanding of the therapy’s impact on patient outcomes and resource use, and guide future use of this approach.

## The way forward

As for any evolving treatment modality, it will take concerted effort and the alignment of multiple factors for radioligand therapy to be properly integrated into cancer care. A shift towards multidisciplinary working and re-evaluation of discrete, independent health specialties is essential—not just for radioligand therapy but for all cancer treatment.

The report proposes 10 key actions to be taken to help overcome existing barriers (Table 1). These will require concerted action by multiple stakeholders including decision-makers, nuclear medicine professionals and the broader cancer clinical community, hospital managers, patient organisations, researchers and industry.

**Table 1:** Key actions to overcome the barriers to greater integration of radioligand therapy in clinical cancer care

Barrier	Action needed
Low awareness and understanding	• Increase awareness of radioligand therapy and the role of nuclear medicine among decision-makers, people with cancer and the clinical cancer community.
Limited professional capacity, training and workforce planning	• Harmonise education and training standards across Europe for nuclear medicine specialists and all members of the multidisciplinary cancer team. • Ensure that nuclear medicine specialists have adequate capacity to participate in multidisciplinary cancer care processes.
Unclear models of care	• Develop clear processes and patient pathways for care in each national context.
Inadequate physical capacity and resourcing in hospitals	• Ensure adequate hospital capacity and resources for delivery of radioligand therapy to meet current and future demand.
Evolving legislation, regulation and policy	• Incorporate radioligand therapy into national, regional and local cancer plans. • Establish clear, consistent regulatory frameworks for the use of radioisotopes spanning approval, funding and reimbursement. • Ensure continued supply and appropriate disposal policies.
Lack of data and research	• Invest in real-world data on radioligand therapy to better understand patient outcomes and cost-effectiveness. • Identify and share best practices to optimise and standardise care.

## Conclusions

Radioligand therapy may provide life-enhancing treatment for patients with cancer who have limited therapeutic options. To ensure it can be appropriately embedded into cancer care, greater integration of nuclear medicine into models of care, workforce planning and hospital resourcing is needed [14, 15, 26]. At a policy level, the inclusion of radioligand therapy in cancer-related policies and future cancer plans—such as Europe’s Beating Cancer Plan—will also be essential, as will be proactively addressing healthcare system capacity issues to enable the provision of safe, high-quality care for both inpatients and outpatients across settings.
